# Dr. Harris’ Case of Corneous Excrescence on the Leg

**Published:** 1842-07-30

**Authors:** C. H. Harris


### MEDICAL EXAMINER.

NEW SERIES.

No. 31.] PHILADELPHIA, JULY 30, 1842. [Vol. I.

*Case of Corneous Excrescence on the Leg.* By C. H. Harris, M. D.

To the Editors of the Medical Examiner.

Gentlemen,—Believing that such growths of the cuticle as are here reported, are exceedingly rare, I have thought proper to transmit to you for publication in your very valuable journal, if you deem it worthy of a place, the history, as well as the treatment of this case.

Very truly yours,

C. H. Harris.

*Buckingham county, (Va.) July* 20, 1842.

In June, 1841, I was requested by a Mr. T. S. to visit a negro who, to use the gentleman's own language, had a horn growing from her leg. Upon reaching the house (in company with my brother, Dr. G. W. H.) the negro was shown us, and upon examination we found on the outer side of the right leg, just below the knee, a large projection, evidently an increased growth of the cuticle or epidermis, and resembling, more than any thing that I can compare it to, a ram's horn. It was two and a half or three inches in diameter at its base, and from six to eight inches in length, and was void of all sensibility. Upon my trimming it with my knife, she did not make the least complaint. Her general health appeared good. Upon inquiry, we ascertained that this girl (about twenty or twenty-one years of age) had had this limb, two or three years previously, very much burnt, and shortly afterwards this unnatural growth made its appearance.

Upon consultation, we at once came to the conclusion that this horn (if I may so term it) should be removed, and the system put under the influence of some remedy calculated to obviate this perverted growth. I suggested arsenic. The operation would have been performed at this time, but on account of the absence from home of the white family at the time of our visit, we concluded it would be best to defer it, as no inconvenience could arise from a short postponement. This negro resided in Goochland county, and being myself compelled to leave soon afterwards for Buckingham county, my place of residence, the case was left in the hands of my brother and his partner in the profession, Dr. J. W., and sometime during the next month (July) it was removed by these two gentlemen.

I have not seen the negro since the removal of this horn, but understand from her mistress that the place from which it was taken has not yet entirely healed; and at different times there has been an effort on the part of the disease to return; but before obtaining much growth, it fell off of its own accord. Her general health is still good.

After the operation, her system was put under the influence of no internal remedy, and I think it is to be regretted that this was not done; in fact, it would not now be too late to try what effect arsenic, or some other remedy, would exert upon this disease.

The specimen I now have in my possession.

